# Supplementary material for: Variation in waiting times by diagnostic category: an observational study of 1,951 referrals to a neurology outpatient clinic
Source: BMJ Neurol Open. 2021 Jun 3;3(1):e000133. doi: 10.1136/bmjno-2021-000133 (PMC8183200; doi:10.1136/bmjno-2021-000133)
Supplement: Supplementary data [file bmjno-2021-000133supp001.pdf]

1 Supplementary Materials

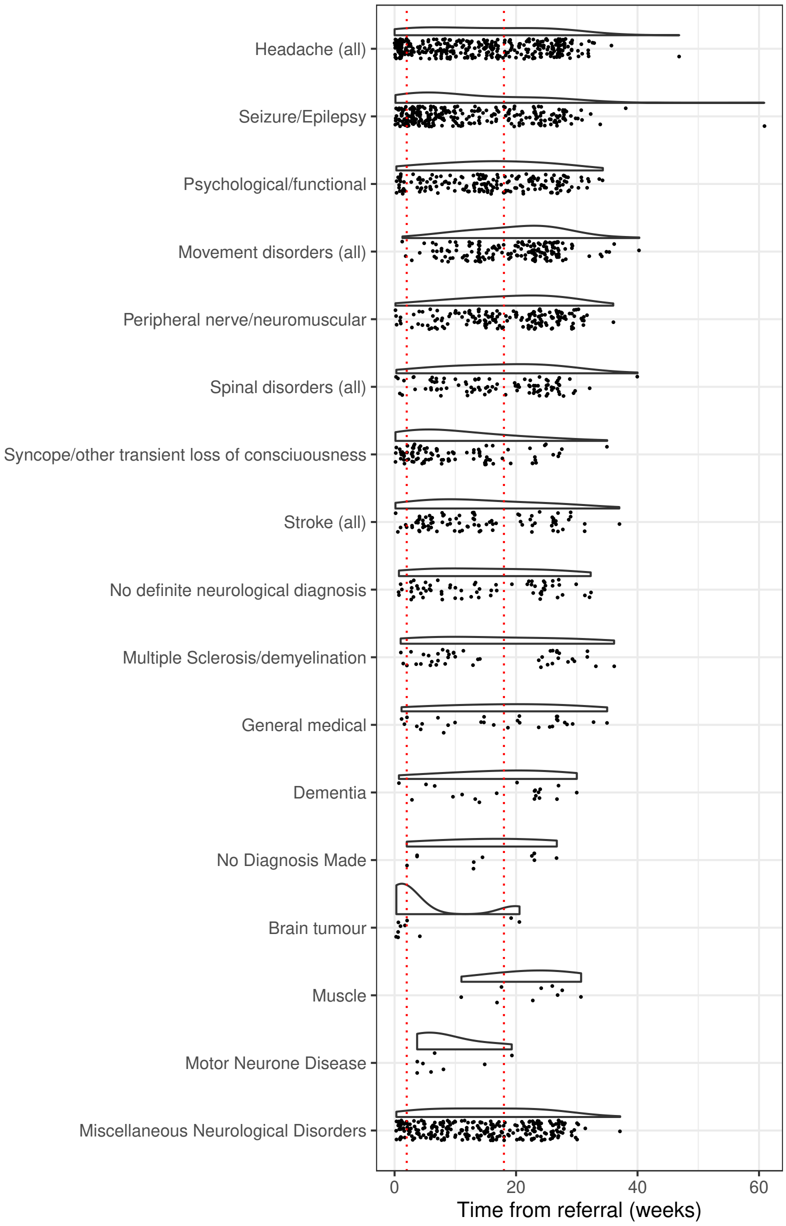

Figure 1: Distribution of waiting time from referral to new appointment including outliers and small categories.

Biggin F, *et al. BMJ Neurol Open* 2021; 3:e000133. doi: 10.1136/bmjno-2021-000133

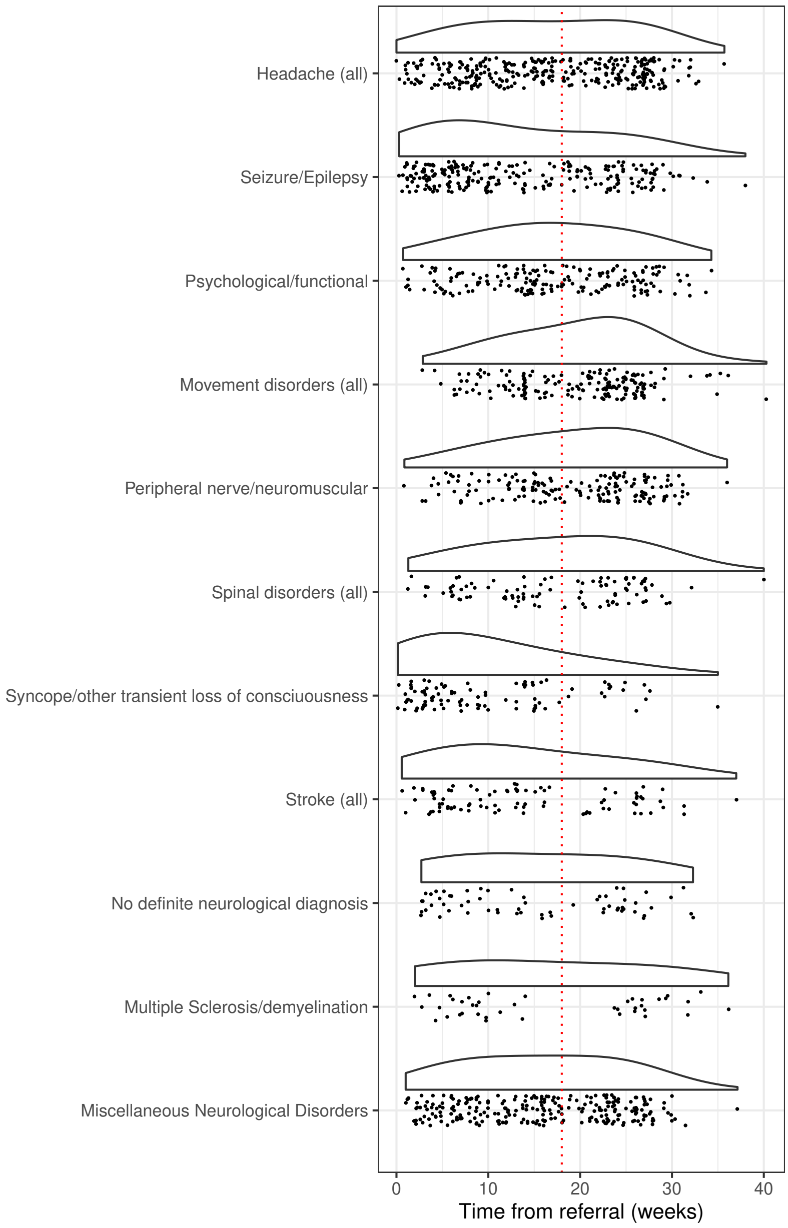

Figure 2: Distribution of waiting time from referral to new appointment after removing patients referred on a 2 week pathway for suspected CNS cancer or a first seizure.

Biggin F, *et al. BMJ Neurol Open* 2021; 3:e000133. doi: 10.1136/bmjno-2021-000133

	Seizure/Epilepsy	Psychological/ Functional	Movement Disorders	Peripheral nerve/ neuromuscular
Headache (all)	2.0×10^{-5} *	0.15	$1.3 \times 10{-}4$ *	$4.4 \times 10{-}3$
Seizure/Epilepsy		$1.9 \times 10{-}7$ *	$3.6 \times 10{-}14$ *	$2.0 \times 10{-}10$ *
Psychological/Functional			$3.3 \times 10{-}3$	0.05
Movement Disorders				0.41

Table 1: P-values from pairwise Kolmogorov-Smirnov tests of the empirical cumulative distributions of waiting time for the 5 most common diagnostic categories. *indicates statistical significance at alpha = 0.05 using Bonferroni corrections for multiple testing.

Biggin F, *et al. BMJ Neurol Open* 2021; 3:e000133. doi: 10.1136/bmjno-2021-000133
